# 'Live more': Study protocol for a community-based lifestyle education program addressing non-communicable diseases in low-literacy areas of the South Pacific

**DOI:** 10.1186/s12889-015-2560-1

**Published:** 2015-12-09

**Authors:** L. M. Kent, P. Reierson, D. P. Morton

**Affiliations:** Lifestyle Research Centre, Avondale College of Higher Education, 582 Freemans Drive, Cooranbong, NSW 2265 Australia; International Programs, Adventist Development and Relief Agency (ADRA) Australia, Wahroonga, NSW 2076 Australia

**Keywords:** South Pacific, Reflect, CHIP, Non-communicable diseases, Lifestyle intervention, Low-literacy

## Abstract

**Background:**

Non-communicable diseases (NCDs) have reached epidemic proportions in Pacific Island countries. Unhealthy lifestyle is one of the major risk factors and lifestyle interventions have been shown to be efficacious for primary, secondary and early tertiary prevention. However, there is a paucity of evidence regarding effective community-based lifestyle interventions in the Pacific Islands. The Complete Health Improvement Program for high-income countries was contextualised for rural communities with relatively low-literacy rates in low-income countries using the REFLECT delivery approach. This study will assess the effect of this ‘Live More’ program to reduce participant’s NCD risk factors and improve lifestyle behaviours associated with health and wellbeing, in low-literacy communities in countries of the South Pacific.

**Methods/Design:**

This study is a 6-month cluster-randomised controlled trial of 288 adults (equal proportions of men and women aged 18 years and over) with waist circumference of ≥92 cm for men and ≥80 cm for women in four rural villages in each of Fiji, Vanuatu and Solomon Islands. Participants will permanently reside in their village and be able to prepare their own meals. Two villages will be randomised to the ‘Live More’ intervention (*n* = 24) or to control receiving only country specific Ministry of Health literature (*n* = 24). Intervention participants will meet three times a week in the first month, then once a week for the next two months and once a month for the last three months. Themes covered include: NCDs and their causes; and the benefits of positive lifestyle choices, positive psychology, stress management, forgiveness and self-worth, and how these influence long-term health habits. Outcome assessments at baseline, 30-days, 3-months and 6-months include body mass index, waist circumference, blood lipids, blood pressure and blood glucose. Secondary outcomes include changes in medication and substance use, diet, physical activity, emotional health and supportive relationships, collected by lifestyle questionnaire at the same time points.

**Discussion:**

This is the first lifestyle intervention using the Reflect approach to target NCDs. The findings from the study will be used to guide broader delivery of a lifestyle intervention to improve health and wellbeing across the South Pacific.

**Trial registration:**

Australian New Zealand Clinical Trials Registry ACTRN12614001206617.

## Background

Until recently, non-communicable diseases (NCDs), also referred to as ‘chronic diseases’, were viewed as a problem of high-income countries while communicable diseases were the major threat to health in Low and Middle Income Countries (LMICs). However, on a global scale the shift from communicable to NCDs as the major cause of mortality [1] is now complete, with NCDs being responsible for 63 % of all deaths worldwide [2]. In fact, the impact of NCDs is now felt most strongly in LMICs [3] which bear 86 % of the global burden of premature deaths from NCDs [4]. Further, many LMICs now face a double burden of disease from communicable and non-communicable risks [5, 6] resulting in significant social disadvantage and economic burden on individuals, families and society at large [3]. It is projected that as a consequence of NCDs LMICs will accumulate economic losses of US$7 trillion over the next 15 years and produce millions of people trapped in poverty [4].

The four main NCDs are cardiovascular diseases (CVD), cancer, chronic respiratory diseases and diabetes. Diabetes in particular is increasing to epidemic proportions, with 387 million people in the world living with the condition and it was responsible for 4.9 million deaths in 2014 [7]. Eighty percent of people with diabetes live in LMICs, and the Western Pacific (which includes the South Pacific) is seen as the epicentre of the diabetes epidemic [7]. The situation in the South Pacific Islands is particularly alarming with seven of the top ten countries in the world with the highest prevalence of diabetes being Pacific Islands [7]. In Vanuatu, almost 1 in 4 (24 %) of the population have diabetes, and there are similar trends in other Pacific Island countries [7].

The urgency and need to address NCDs in LMICs through appropriate prevention and treatment has only recently been recognised by the United Nations (UN), governments and the non-government organization (NGO) sector [3]. At the 42nd Pacific Islands Forum Communique in 2011, it was declared that ‘NCDs have reached epidemic proportions in Pacific Island countries and territories and have become a human, social and economic crisis requiring an urgent and comprehensive response’ [3]. Clearly, there is a need for effective and relevant programs to address NCDs, particularly in the South Pacific [3, 4]. Unhealthy lifestyle is one of the major risk factors of NCDs [8] and lifestyle interventions have been shown to be efficacious for their primary, secondary and early tertiary prevention [9–13]. However, there is a paucity of evidence regarding effective community-based lifestyle interventions in the Pacific Islands [14].

The Complete Health Improvement Program (CHIP) is a community-based lifestyle intervention that originated in the United States of America and has demonstrated significant benefits for the management of cardiovascular disease [13, 15, 16], type 2 diabetes mellitus [13] and depression [17, 18]. The positive health outcomes have been documented and published in more than 25 peer reviewed journals [19]. CHIP is a holistic, evidence-based, education program which addresses various aspects of health including nutrition, physical activity, substance use, stress, self-worth and happiness [19]. The CHIP intervention involves 18 one to 1.5-h group sessions over 6 to 12 weeks.

While the program was developed for middle to high socio-economic (SES) groups in the United States, recent studies from other high-income countries, such as Australia/New Zealand and Canada [20, 21] have shown similar levels of effectiveness, despite the inherent cultural differences. In addition, CHIP delivered to free-living residents of Appalachia, a lower SES community in the Athens County which struggles with the highest poverty levels in Ohio, produced similar results to those observed in higher SES groups [22].

There have been no published studies of the effectiveness of the CHIP intervention in the Pacific Islands to date, however, unpublished data from large urban centres of the Solomon Islands, Vanuatu and Fiji shows positive biometric outcomes following the program. Noting these pilot findings and the considerable literature supporting the effectiveness of the CHIP intervention, an Australian NGO, the Adventist Disaster and Relief Agency (ADRA) Australia, sought to use the CHIP intervention to address the high rates of NCDs in the Pacific Islands. However, it was recognised that the program was less suited to the rural and most marginalised parts of the Pacific Islands, as well as other LMICs, because: to engage with the program required English literacy, the content was not culturally identifiable, ingredients for suggested recipes were not available, and the program required technical equipment that was not commonly accessible such as video projection. As a result, a contextualised version of the CHIP intervention, appropriate for rural communities with relatively low literacy rates, was developed. The program communicated the core messages embedded in the conventional CHIP but also focused more widely on food security, availability, access and utilisation.

The developed program was built on a model of adult learning and social change known as Regenerated Freirean Literacy through Empowering Community Techniques (REFLECT). The REFLECT model fuses the theories of Brazilian educator Paulo Freire with Participatory Methodologies (PMs) and was developed in the 1990s to link adult literacy to empowerment [23]. Today, the REFLECT approach is used by over 500 organisations in more than 70 countries worldwide [24]. REFLECT utilises a “Reflect Circle” which is a group methodology in which the teacher facilitates discussion, rather than lectures to a ‘class’. The groups meet regularly to discuss local issues and how these can be addressed, while at the same time developing literacy skills. Key to the REFLECT approach is creating a space where people feel comfortable to meet and discuss issues relevant to them and their lives. This is often the first opportunity the village members have to conduct a systematic analysis of their lives and environment. While this process may lack in technical expertise, it is enriched by the local specificity and the connections and insights that emerge, which would not be elucidated by a more formal education process.

ADRA has been using the REFLECT approach to enact social change, financial security and literacy/numeracy in countries such as Tanzania, Albania, Sudan and Cambodia. Anecdotally, these programs have resulted in perceived health and wellbeing benefits, despite not specifically addressing these domains. Hence, it was rationalised that the REFLECT approach might be appropriate for delivering a program specifically targeting NCDs.

This paper provides the rationale and methods for the study of the adapted version of the CHIP intervention, contextualised for low SES communities in the islands of the South Pacific, utilising the REFLECT approach of delivery. The program is referred to as ‘Live More’ and is designed to reduce participant’s NCD risk factors and improve lifestyle behaviours associated with health and wellbeing.

## Methods/Design

### Study design

**Fig. 1:**
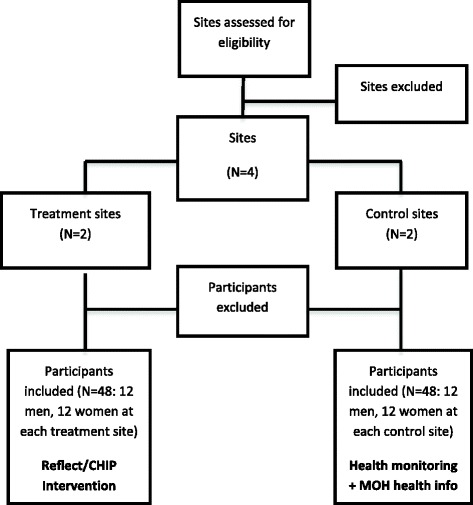
Study design and flow

The ’Live More’ program will be evaluated using a cluster randomised controlled trial. The study design is illustrated in Fig. 1. The 6-month intervention will be conducted in three Pacific Island countries: Solomon Islands, Vanuatu and Fiji. In each country there will be two intervention villages and two control, resulting in a total of six intervention villages and six control villages across the three countries. Assessments will be conducted at baseline, and repeated at 30 days, 3 months and 6 months post baseline. The design, conduct and reporting of this study will adhere to the Consolidated Standards of Reporting Trials (CONSORT) guidelines for group trials.

### Ethical approval

The study was approved by the (name of the ethics committee removed for blinding) Human Research Ethics Committee in New South Wales, Australia (ID: 2014/03) on 18th of March 2014. The protocol for the study is registered with the Australian New Zealand Clinical Trials Registry (ID: ACTRN12614001206617).

### Setting

The local ADRA supervisor will meet with the chiefs/community elders of rural/semi-rural villages within 2 h drive of Honiara in the Solomon Islands, Port Vila in Vanuatu and Suva in Fiji to outline the program and gauge interest.

### Village eligibility criteria and randomisation

A village will be eligible to participate in the study if there is:

1. an expressed endorsement and positive commitment by the village chief/community leader;
2. observable overweight in the community (estimation of at least 40 % as observed by the ADRA Supervisor);
3. relatively low literacy rate/education level in the potential participants as indicated by the community leader;
4. adequate interest by residents who meet the participant eligibility criteria (described below) to achieve the required cluster sample size (described below);
5. willingness to participate in the study as either a treatment or control site.

Once eligible villages are identified, ensuring they are not in immediate proximity to each other to avoid cluster contamination, the villages in each country will be randomised to either intervention or control by a researcher not familiar with the geographic location or nature of the village. Figure 1 shows the sequence for allocation of sites to treatment and control conditions.

### Participant recruitment and eligibility criteria

Participants will be recruited through a village meeting called by the village elder. During this meeting, an ADRA Supervisor together with a designated health professional (HP-doctor or nurse) will provide information about the program. Following the information session, a call for volunteers who meet the eligibility criteria will be made. To be considered eligible to participate in the study the individuals will:

1. be 18 years of age or older;
2. live permanently in the village (for the duration of the program)
3. have a waist circumference of ≥92 cm for men and ≥80 cm for women (as levels at and above these are indicative of risk of NCD) [25];
4. be prepared to engage in the ‘Live More’ intervention and monthly post-program meetings (total duration of 6 months);
5. be able to provide their own meals (because this will enable compliance with the program’s dietary recommendations).

An equal number of males and females will be recruited for the study. No payments will be requested of participants and the participants will not receive any financial remuneration for their involvement.

Individuals will be considered ineligible to participate in the study if they meet one or more of the following exclusion criteria:

1. unstable angina;
2. myocardial infarction within the previous 12 months;
3. coronary by-pass surgery within the previous 12 months;
4. other medical contraindications for dietary change or increased physical activity, as determined by the HP;
5. chronic infectious disease/s, as participants illness may affect participation in the program;
6. are a direct relative of the village chief (to reduce bias in recruiting).

All eligible participants will be explained the consent process, and informed that all personal information will be confidential and anonymous. They will then be asked to sign or make their mark on the consent form or give the appointed HP for the study verbal consent to sign on their behalf in the presence of a witness (in case of literacy constraints).

### Power calculation

Data from CHIP interventions conducted in Australia and New Zealand [20] were used for the power analyses. Power calculations were conducted on all the biometrics to be measured in the present study, which indicated that of all the biometrics, detecting a significant change in triglycerides would require the greatest sample size. Using an estimated mean baseline triglyceride level of 1.15 mmol/L with a standard deviation of 0.33 mmol/L, to achieve a 5 % level of significance with 80 % power will require 87 participants in each of the three countries. Allowing for a 10 % loss to follow up at 6 months, in each country a total of 96 participants will be recruited, constituting two intervention villages each with 24 participants and two control villages each with 24 participants. In total, 288 participants from the three countries will be involved in the study: 144 intervention and 144 control.

### Program facilitators

Community leaders of the respective intervention villages will propose one eligible candidate of each gender from the local village to act as facilitators of the program. To be considered eligible the potential facilitator must: have completed secondary education, be fluent in English, be respected in the village, be a non-smoker, and have an interest in health and desire to positively impact the health of their village. ADRA Australia will provide the local village facilitators with two weeks of training using Strength Based [26–28] and Appreciative Inquiry (AI) approaches. The training sessions will cover the principles of the CHIP intervention and the REFLECT methodology, as well as the development of facilitation skills and the research methods used in the study.

Following the training, the local ADRA supervisor together with the facilitators from the study villages will collaborate with ADRA Australia and the local ADRA office to ensure a Facilitators Manual (including 18 lesson plans) meet the local context. Following the 6-month program, this collaborative process will be repeated to further contextualise the Facilitator Training and Process Manual’s for the Pacific. The local ADRA supervisor will be responsible for follow up, mentoring the village facilitator and monitoring the program and research.

### Intervention

Participants will receive 6 months of the contextualised 18 session ‘Live More’ program which is based on and follows the order of the 18 session ‘Westernised’ CHIP intervention, which has been previously described [19]. Themes covered include: the causes of NCDs; the benefits of positive lifestyle choices, particularly diet and physical activity as therapy for conditions including obesity, type 2 diabetes, and CVD; and positive psychology and how this influences long-term health habits, as well as stress management, forgiveness, and self-worth.

Participants will meet three times a week in the first month, then once a week for the next two months, followed by once a month for the next three months. The specific content of the ‘Live More’ sessions is outlined in Table 1.

**Table 1:** Time line and specific content of the 'Live More' program

Week	Session
0	Baseline biometric measurement, completion of lifestyle questionnaire and demographics
1	1. ‘The Rise and Rise of Chronic Disease’. 2. ‘Lifestyle is the Best Medicine’. 3. ‘The Common Denominator of Chronic Disease’.
2	4. ‘The Optimal Lifestyle’. 5. ‘Eat More, Weigh Less’. 6. ‘Fiber, Your New Best Friend’.
3	7. ‘Disarming Diabetes’. 8. ‘The Heart of the Matter – Heart Health’. 9. ‘Controlling blood pressure and discovering protein’.
4	10. ‘Bone Health Essentials’. 11. ‘Cancer Prevention’. 12. ‘How to grow a family garden. What to grow and when?’
5	30-day biometric measurement, completion of lifestyle questionnaire and feedback. Discussion on understanding results and taking action (with health professional), followed by ‘Circle’ feast/celebration (meal preparation using ‘Live More’ principles learned in earlier sessions).
6	13. ‘Become what you believe and your DNA is not your destiny’.
7	14. ‘Practicing forgiveness’.
8	15. ‘Re-engineering your environment’.
9	16. ‘Stress-relieving strategies’.
10	17. ‘Fix how you feel’.
11	18. ‘From Surviving to Thriving’
12	3-month biometric measurement, completion of lifestyle questionnaire and feedback.
24	3-month biometric measurement, completion of lifestyle questionnaire and feedback.

Each session will typically involve meeting in the ‘Reflect Circle’ where all participants have the opportunity to participate. The session will commence with the facilitator welcoming the participants in a culturally appropriate way and ensuring no-one feels excluded. Participants will be encouraged to discuss their progress and ask any questions that have arisen since the previous session. Using the lesson plan, the predetermined participatory technique and the necessary aids, the facilitator will introduce the theme for the session by exploring what the participants already know about themselves and their local situation. In particular, graphics/visuals in the form of maps, pictures, calendars and matrices will be utilised to describe their current situation and understandings. Following this activity, the participants will be further drawn into the session’s topic by use of a trigger picture, where they will be asked to describe what they see. Throughout this discussion, information will be shared with the group to enhance their understanding of the topic. When all material included in the lesson plan is covered, the facilitator will guide the participants into an action plan to incorporate the learnings from that session into their lives (e.g. how to incorporate 10,000 steps into their day). The session will conclude with congratulations for success so far in the health journey and a reminder of the time and venue for the next session. This whole process empowers the participants to openly discuss their situation and develop strategies to address the issue introduced in that session.

At the end of the 6-months, there will be another biometric measurement, completion of the lifestyle questionnaire and personal feedback. A group feedback session will follow where the achievements of the groups will be acknowledged and graduation certificates distributed. The celebrations will culminate with a ‘Live more’ friendly feast.

### Control villages

In each of the control villages printed health education material developed by the local Ministry of Health will be presented at the first health screening. At each subsequent assessment point, the HP will invite the participant to ask any health related questions from the health literature that was provided at baseline.

### Outcome measures

Data on biometrics, blood measures and health behaviour will be collected on individuals in the intervention and control villages at program entry (baseline), one month, three months and six months by a team of HP’s. A medical assessment, including personal and family health history will be collected at program entry only. The following measurements will be collected:

1. Height, weight, body mass index (BMI), waist circumference and blood pressure will be measured on site by a HP. Weight (in light clothing with shoes removed), to determine BMI, will be measured by a HP, following 12 h fast using commercial weight scales. Blood pressure will be measured using a medical sphygmomanometer by a HP. Measurements will be repeated at each time point using the same equipment to reduce systematic measurement error.
2. Fasting lipid profile and plasma glucose will be drawn on site. Whole blood samples will be collected into capillary and heparinised tubes by the designated HP following via finger prick method. Samples will be removed from the tube for analysis, using the CardioChek PA portable blood analyser within 5 min of the blood draw. All alcohol swabs, lancets, used blood tubes and CardioChek pads will be deposited in biohazard material bags and taken by the HP to the local pathology laboratory or public hospital for incineration.
3. Physical activity will be quantified using a pedometer supplied to the participants who will record their daily steps taken in a log book provided. For illiterate participants, the final reading at the end of each day will be recorded by someone with literacy skills from the same village.
4. Health related behaviours will be assessed using a lifestyle questionnaire administered verbally by the HP (requirement due to the relatively low levels of literacy of the participants). A medical doctor or nurse will be present at the beginning of the project to collect and record information in private. The information will then be given to the ADRA supervisor who will keep it in a locked file.

- Lifestyle habits include: smoking, alcohol, kava and betel nut usage; frequency of daily consumption of limited key foods including, fruit and vegetables, fatty and processed foods, meat and wholegrains/cereals; frequency of weekly light, moderate and strenuous physical activity
- Subjective wellbeing include: emotional health, supportive relationship
- Medical history, including personal and family history of heart problems, high blood pressure and blood glucose, cancer and breathing problems.
- Medication usage. During the initial medical assessment, the participants will present all medications they are taking and the type and dosage will be recorded by the HP.

### Process evaluation

Unlike ‘Outcome evaluation’, as outlined above, which assesses the effectiveness of the program to impact the health of the participants, ‘Process Evaluation’ gives an understanding of *how* the outcome was achieved.

Process data regarding program operations, implementation and delivery will be collected to complement the outcome data. Process measures will include:

1. Participant attendance at sessions. Participants will be deemed to have completed the program if they attend 75 % of the sessions (i.e. 14 of the 18 ‘Live More’ sessions);
2. Facilitator self-efficacy and competency survey. The facilitators will respond to a range of likert items relating to their perceived: level of skill and confidence in program delivery, interest in health before and after their training, and impact on the health of the participants. The facilitators will also be asked in a semi-structured interview about factors enabling their continued engagement and strategies used to engage group participation and attendance;
3. Participant focus group discussion. A minimum of 5 participants from each village will be asked to relay their most significant change during the program. Most Significant Change Stories (MSC) methodology [29] will be used to tease out the participants’ perceived change and benefits from the program;
4. Participant satisfaction with all components of the intervention will be assessed by semi-structured interview to inform the future development of the intervention.

### Statistical methods

The (biomedical) data will be analysed using IBM™ Statistics (version 21). Continuous data will be expressed as number, mean, standard deviation (SD). Categorical data will be summarized as counts and percentages. For each country, differences between participants in the intervention and control groups at baseline will be examined using Chi square for categorical variables and independent samples t-tests for continuous variables. The extent of changes (percent, and mean with 95 % confidence intervals (CI)) from baseline, one month, three months and six months will be assessed using Analysis of Variance (repeated measures). For all analyses, results are considered significant at *P* < 0.05.

The qualitative data obtained through the various participatory, qualitative techniques listed above, will first be checked for accuracy with some of the participants and then with program staff (facilitators and supervisor). For the analysis, focus group data from the first village will be broken down into parts and examined for emergent categories (themes or patterns) and sub-categories, then coded. These categories and sub-categories may at this stage start to show a theory. Coding of the second focus groups will be done with the first in mind. Subsequent focus group discussion in the other two countries will also be coded with the emerging theory in mind.

### Discontinuance of the research

Provision is made for the research to cease, at a village and/or country level, in the event that the successful completion of the project is jeopardised by:

1. natural disaster,
2. civil unrest
3. irretrievable communication breakdown with village leaders
4. in-country mismanagement of funds.

## Discussion

In this paper we described the rationale and study protocol for the’Live More’ lifestyle intervention targeting NCDs within villages in the South Pacific. To the authors’ knowledge,’Live More’ is the first lifestyle intervention using the Reflect approach targeting low-income countries, such as those found in the South Pacific.

Targeting low-income countries in the South Pacific is important because increased access to nutritiously poor imported foods, coupled with limited funds available to support the delivery of public health campaigns and health care services, are having deleterious health consequences [30]. Over the last two decades, poverty has been growing in some countries of the South Pacific, with Solomon Islands and Vanuatu now on par with Guinea, Burundi, Senegal and Bangladesh [29]. Indeed awareness of the association between NCDs and poverty is growing with the NCD Alliance, consisting of four international NGO federations, advocating for the recognition that NCDs are a major cause of poverty, a barrier to economic development and a global emergency [31].

While there is strong evidence that lifestyle interventions are effective in addressing NCDs in high-income countries [9–12], peak bodies such as the World Health Organization acknowledges that there are few evidence-based lifestyle interventions suitable for low SES communities [32]. More specifically, regarding the countries in this study, these interventions are sparse in Fiji, and non-existent in Vanuatu and the Solomon Islands [16]. Furthermore, where programs do exist, these are costly and have limited usefulness in the communities that have low literacy levels and limited access to technology.

Interventions that provide professional learning opportunities for facilitators who live with and can mentor participants in the same village may provide a valuable framework for sustainable practice. Utilising villagers as facilitators not only uses local resources but can also improve psychological well-being, self-efficacy and confidence, particularly where underemployment is prevalent [33]. In addition, community self-help that involves the whole community through the interest and awareness it creates can also contribute to its development as a whole [33].

The ’Live More’ program is an innovative intervention targeting participants at risk of NCDs in low-income communities. The strengths of this study include the opportunity to build self-sufficiency and autonomy within a community, the objective measures of various biometrics and PA, and the comprehensive multi-component intervention. The findings from the study will be used to guide delivery of a lifestyle intervention to improve health and wellbeing in the South Pacific.

## Abbreviations

NCD: Non-Communicable Disease
LMIC: Low and Middle Income Countries
BMI: Body Mass Index
CVD: Cardiovascular Disease
UN: United Nations
NGO: Non-Government Organisation
ADRA: Adventist Disaster and Relief Agency
CHIP: Coronary Health Improvement Program
SES: Socioeconomic Status
REFLECT: Regenerated Freirean Literacy through Empowering Community Techniques
PM: Participatory Methodology
CONSORT: Consolidated Standards of Reporting Trials
MSC: Significant Change Stories
AI: Appreciative Inquiry
HP: Health Professional
SD: Standard Deviation
CI: Confidence Interval
